# Human Cardiac Fibroblast Number and Activation State Modulate Electromechanical Function of hiPSC-Cardiomyocytes in Engineered Myocardium

**DOI:** 10.1155/2020/9363809

**Published:** 2020-07-16

**Authors:** Cassady E. Rupert, Tae Yun Kim, Bum-Rak Choi, Kareen L. K. Coulombe

**Affiliations:** ^1^Center for Biomedical Engineering, School of Engineering and Division of Biology and Medicine, Brown University, Providence, RI, USA; ^2^Cardiovascular Research Center, Rhode Island Hospital and Alpert Medical School of Brown University, Providence, RI, USA

## Abstract

Cardiac tissue engineering using hiPSC-derived cardiomyocytes is a promising avenue for cardiovascular regeneration, pharmaceutical drug development, cardiotoxicity evaluation, and disease modeling. Limitations to these applications still exist due in part to the need for more robust structural support, organization, and electromechanical function of engineered cardiac tissues. It is well accepted that heterotypic cellular interactions impact the phenotype of cardiomyocytes. The current study evaluates the functional effects of coculturing adult human cardiac fibroblasts (hCFs) in 3D engineered tissues on excitation and contraction with the goal of recapitulating healthy, nonarrhythmogenic myocardium in vitro. A small population (5% of total cell number) of hCFs in tissues improves tissue formation, material properties, and contractile function. However, two perturbations to the hCF population create disease-like phenotypes in engineered cardiac tissues. First, increasing the percentage of hCFs to 15% resulted in tissues with increased ectopic activity and spontaneous excitation rate. Second, hCFs undergo myofibroblast activation in traditional two-dimensional culture, and this altered phenotype ablated the functional benefits of hCFs when incorporated into engineered cardiac tissues. Taken together, the results of this study demonstrate that human cardiac fibroblast number and activation state modulate electromechanical function of hiPSC-cardiomyocytes and that a low percentage of quiescent hCFs are a valuable cell source to advance a healthy electromechanical response of engineered cardiac tissue for regenerative medicine applications.

## 1. Introduction

Tissue engineering approaches to cardiac modeling, therapeutic development, and regeneration are promising but have been hindered in part by the electromechanical immaturity of human pluripotent stem cell- (hPSC-) derived cardiomyocytes. This immaturity has been documented in cardiovascular research from the single-cell level, where electrophysiological patch-clamp recordings have revealed a reduced inward rectifier potassium current (*I*_K1_) [1], expression of pacemaker channels to cause frequent spontaneous contractions [2], and low expression of L-type channels [3], to implantation in animal models, where arrhythmia and insufficient coupling are reported with the injection of cells [4, 5] and engineered tissues [6–8], respectively. The ubiquity of these obstacles necessitates further exploration of how a hPSC-cardiomyocyte electromechanical phenotype develops in response to differentiation protocols [9, 10], culture conditions, and heterocellular interactions, to name only a few. Noncardiomyocytes have been shown to be necessary for achieving optimal mechanical performance of engineered cardiac tissues [11], which makes the study of heterocellular interactions particularly invaluable to the cardiovascular engineering field.

As thoroughly summarized by Zhou and Pu [12], the ratio of cardiomyocytes to noncardiomyocytes in the heart varies widely depending on the methodology of measurement. However, studies of the human ventricle come to a general consensus that cardiomyocytes contribute to about one-third of the tissue by cell number [13, 14], with endothelial cells and cardiac fibroblasts comprising the majority of noncardiomyocytes. A variety of human cell types have been used to replace this noncardiomyocyte population in engineered tissues ranging from mesenchymal stem cells and umbilical vein endothelial cells [15–17] to brain pericytes [18] and neonatal dermal fibroblasts [11, 19], and simply relying on the noncardiac population resulting from the differentiation process [11]. These support cell types provide functional benefit to engineered tissues including improved extracellular matrix (ECM) production and contractile force. However, they do not take into account native myocardial cellular heterogeneity, which provides a physiologically relevant list of potential support cell candidates that may also impact cardiac function.

Among these candidates are cardiac fibroblasts (CFs), which comprise a significant portion of the heart by cell number [12]. Fibroblasts of the cardiac lineage in particular are critical for providing structural organization and support in homeostasis and are prominent modulators of acute and chronic cardiac injury [20]. In the healthy heart, CFs produce and maintain the ECM, specifically fibronectin, laminin, and collagens I, III, and IV [21–23]. In a disease state, CFs undergo activation to a myofibroblast phenotype, characterized by increased proliferation, contractility, and extracellular matrix production [24]. Myofibroblasts express gap junction proteins connexin 43 and 45, and at a low concentration, they speed conduction and upstroke velocity in vitro [25, 26]. Recent studies also suggest that myofibroblasts electrically couple to cardiomyocytes at the border zone of infarcted murine hearts [27, 28]. Their active participation in myocardial structural maintenance and disease makes hCFs an excellent candidate for developing engineered cardiac tissues with more mature functionality while incorporating a physiologically relevant toolkit to model cardiac injury, aging, and fibrosis.

In this study, we demonstrate that adult human cardiac fibroblasts (hCFs) can be used to optimize engineered cardiac tissue electromechanical performance and that altering culture conditions produces arrhythmogenic phenotypes based on percentage and preconditioning of the hCF population. We demonstrate that, in our hands, neonatal human dermal fibroblasts (NHDFs) cannot be leveraged in the same way. We show that low hCF inclusion increases contractile force by three-fold compared to hiPSC-cardiomyocyte-only control tissues, while higher percentages increase excitability and spontaneous beating rate. Additionally, we show that serial passaging and the resulting increased myofibroblast activation are at leastpartially retained by hCFs in engineered cardiac tissues, negating the benefit to function that early-passage hCFs provide. We also report and characterize, for the first time, the appearance of contractile alternans—the periodic alternation between full-force and partial-force contractions—and their pacing frequency dependence in engineered tissues. These data demonstrate that hCFs are a valuable cell type for manipulating the pathophysiological phenotype of engineered cardiac tissues and that our tissue engineering platform has the sensitivity to distinguish differences in functional performance in response to heterocellular interactions between hiPSC-cardiomyocytes and hCFs.

## 2. Methods

### 2.1. Cell Culture and Differentiation

Gibco® Human Episomal Induced Pluripotent Stem Cells (hiPSCs) were purchased from Thermo Fisher Scientific. hiPSCs were cultured on plates coated with truncated human vitronectin in Essential 8 Medium (Thermo Fisher Scientific). Small molecule Wnt modulation was used for directed cardiac differentiation, as previously described [29]. In brief, hiPSCs were singularized, seeded on Matrigel-coated plates, and grown to approximately 70% coverage at which point chemically defined differentiation was started. Cells were sequentially treated with 6 *μ*M CHIR 99021 followed by 5 *μ*M IWP2 in RPMI 1640 supplemented with L-ascorbic acid and human serum albumin [10]. After one week of differentiation, cells were maintained in RPMI 1640 supplemented with B27 (RPMI/B27). hiPSC-cardiomyocytes were harvested using 0.25% trypsin in 0.5 mM EDTA and used for engineered tissue experiments at 14–25 days of differentiation.

Healthy adult human ventricular cardiac fibroblasts (hCFs) were purchased from a commercial vendor (63-year-old female, lot #416Z048.9, PromoCell) and maintained in DMEM/F12 supplemented with 10% fetal bovine serum (FBS) and 1% penicillin-streptomycin (pen-strep). hCFs were expanded and used for downstream experiments (passages 3-9, reported with each data set in Results). For 2D myofibroblast substrate stiffness activation experiments, coverslips were coated with polyacrylamide gels at 10% acrylamide and 0.1% bis-acrylamide for a stiffness of approximately 12 kPa [30]. Gels were functionalized with 0.2 mg/mL human fibronectin and seeded with hCFs for 72 hours. For 2D myofibroblast biochemical activation experiments, hCFs seeded on glass coverslips were treated with 10 ng/mL TGF-*β*1 for 48 hours.

Lentiviral GFP labeling of human cardiac fibroblasts was performed using a commercially available lentivirus (Applied Biological Materials). Passage 5 hCFs were incubated with pLenti-GFP-EF1 *α* lentiviral particles at a MOI of 3 in the presence of 5 *μ*g/mL polybrene for 24 hours. hCF^GFP^ cells were purified through the addition of 50 *μ*g/mL neomycin to culture media and expanded for five passages. hCF^GFP^ cells were used at passage 10 in engineered cardiac tissues.

Neonatal human dermal fibroblasts (NHDFs; a gift from Dr. Jeffrey Morgan) were maintained on tissue culture plastic in DMEM supplemented with 10% FBS and 1% pen-strep. Cells were expanded using 0.05% trypsin and used at passage 8.

### 2.2. Immunofluorescence Staining and Imaging

For imaging hCFs in monolayer culture, cells were fixed in 4% paraformaldehyde (PF) for 10 minutes at 4°C. Cells were permeabilized with 0.25% Triton™ X-100 (Sigma-Aldrich) and were incubated in primary antibodies against *α*SMA (1: 100, rabbit, Abcam ab32575) and vimentin (1 : 100, mouse, Sigma-Aldrich V6630). Cells were labeled with fluorescent secondary antibodies Alexa Fluor®488 Goat-anti-mouse (1 : 100, Life Technologies A-11001) and Alexa Fluor®594 Goat-anti-rabbit (1 : 100, Life Technologies A-11012) and counterstained with DAPI. Cells were imaged using a Nikon Ti-E inverted fluorescence microscope, and images were processed and fluorescence intensity quantified using CellProfiler [31].

For imaging engineered cardiac tissues, ECTs were fixed in 4% PF for 10 minutes at ambient temperature before being embedded in paraffin and sectioned at 20 *μ*m thickness. For imaging of myofilaments, a Proteinase K digest was performed for 10 minutes at 37°C (10 *μ*g/mL, Roche 745723) for antigen retrieval followed by incubation overnight in mouse anti-*α*-actinin (1 : 800, Abcam ab9465-500). Sections were then labeled with Alexa Fluor®488 Goat-anti-mouse (1 : 100, Life Technologies A-11001) and counterstained with DAPI. For imaging GFP-labeled hCFs in ECTs, heat-induced epitope retrieval was performed for 10 minutes in a 0.01 M citrate buffer followed by incubation overnight in antibodies against GFP (1 : 1000, rabbit, Abcam ab183734) and vimentin (1 : 100, mouse, Sigma-Aldrich V6630). Sections were then labeled with Alexa Fluor® 594 Goat-anti-rabbit and Alexa Fluor®488 Goat-anti-mouse (1 : 100, Life Technologies A-11037 and A-11001, respectively) and counterstained with DAPI. Sections were imaged using a Nikon Ti-E inverted fluorescence microscope.

### 2.3. Flow Cytometry

Cells for flow cytometry analysis were singularized and fixed in 4% PF for 10 minutes at ambient temperature. Cells were stained with antibodies against cardiac troponin T (cTnT, 2 *μ*g/mL; Thermo Fisher Scientific) and alpha-smooth muscle actin (*α*SMA, 0.5 *μ*g/mL; Abcam). CTnT+ cells were considered cardiomyocytes, *α*SMA+ cells fibroblast like, and double-positive cTnT+/*α*SMA+ immature cardiomyocytes [32]. Samples were run on either an Attune NxT or BD FACSAria Flow Cytometer and analyzed with either FlowJo or Flowing Software. Purity of input hiPSC-cardiomyocyte populations was 50% ± 14% cTnT+ in hCF doping experiments and 53% ± 12% cTnT+ in hCF serial passaging experiments (*n* = 3 and 4, respectively; mean ± SEM).

### 2.4. Quantitative RT-PCR

mRNA was extracted from cells and tissues using the RNeasy Mini Kit, and mRNA concentration was measured with a NanoDrop 1000 Spectrophotometer. CDNA was synthesized from an equal amount of mRNA using the SuperScript III First-Strand Synthesis System. CDNA samples were combined with custom primers (Supplemental Table S1) and SYBR Master Mix, and quantitative real-time PCR was run on an Applied Biosystems® 7900 Fast Real-Time System. Hypoxanthine-guanine phosphoribosyltransferase (HPRT) was used as an internal control of basal transcription rates, and the relative expression compared to control was calculated for genes of interest using the 2^−ΔΔCt^ method [33].

### 2.5. Mold and Tissue Formation

Molds and tissues were formed as previously described [34, 35]. In brief, custom acrylic molds were fabricated using a 100 W CO_2_ laser and polydimethylsiloxane (PDMS) was poured into acrylic negatives and cured at 60°C. Tissues were formed by combining 1 × 10^6^ hiPSC-cardiomyocytes and 0-15% hCFs or 0-20% NHDFs with 2.5 mg/mL rat tail collagen-1 at a 50%/50% vol/vol ratio for a final concentration of approximately 16 × 10^6^ hiPSC-CMs/mL and 1.25 mg collagen/mL. Cell-collagen solution was pipetted into PDMS molds, maintained in RPMI/B27, and stimulated with a 4 ms biphasic pulse at 1 Hz and 5 V/cm for the duration of culture.

### 2.6. Tissue Mechanical Measurements

Mechanical measurements were performed after one week of culture as previously described [35]. Engineered tissues were cut in half, and their passive and active mechanical properties were measured with an ASI 1600A system (Aurora Scientific). Strips were mounted on hooks attached to a 5 mN force transducer and high-speed motor arm, bathed in Tyrode's solution with 5 mM glucose and 1.8 mM CaCl_2_ at 30-34°C, and electrically field stimulated with platinum electrodes. Tissues were stretched from their initial length, *L*_o_ (determined as just above slack length), by 5% steps to 130% *L*_o_. At the final length, tissues were paced with increasing frequency, and the fastest pacing they followed was recorded as the maximum capture rate (MCR).

Calculations were made from the data recorded during mechanical testing to obtain the following values: Active stress, *σ*_a_, was calculated by averaging the active twitch force of 10 contractions and normalizing by the cross-sectional area (CSA). CSA was calculated under the assumptions that tissue height was half the width and cross-sectional shape was an ellipse. Passive stress, *σ*_p_, was calculated by normalizing the passive (baseline) force produced at each step by the CSA, and tissue stiffness (Young's Modulus) was calculated as the slope of the line of best fit of passive stress versus strain at 5–30% strain. Data was processed and analyzed using a custom MATLAB script.

### 2.7. Calcium (Ca^2+^) Imaging

Imaging of tissue-level Ca^2+^ transients was performed simultaneously with mechanical measurements. Prior to imaging, ECTs were incubated in Ca^2+^-free Tyrode's solution with 10 *μ*M Rhod-2 (Life Technologies R1245MP) for 15 minutes at 37°C followed by 15-minute recovery. Tissues were imaged on the TRITC channel of an Olympus IX70 inverted fluorescence microscope during the increased pacing portion of mechanics testing. Video was captured at 30 fps with a Canon EOS 6D DSLR camera, and the data was analyzed using a custom MATLAB script.

### 2.8. Optical Mapping of Action Potentials

ECTs were placed in a temperature regulated chamber (37.0 ± 0.2°C) and perfused with 130 NaCl, 24 NaHCO_3_, 1.0 MgCl_2_, 4.0 KCl, 1.2 NaH_2_PO_4_, 5 dextrose, and 1.25 CaCl_2_, and gassed with 95% O_2_ and 5% CO_2_. ECTs were stained with voltage-sensitive dye, di-4-ANEPPS (Invitrogen), and 5 *μ*mol·L^−1^ blebbistatin was added to the perfusate to reduce the contraction artifact to fluorescence recording. Fluorescence images of APs were recorded from the anterior surface of the heart using a CMOS camera (100 × 100 pixels, 1000 frames/sec, 1.0 × 1.0 cm^2^ field of view, Ultima-L, SciMedia, Japan). Activation maps and APD maps were generated using dF/dt for activation and dF^2^/dt^2^ for repolarization using digital image analysis routines as previously described [36]. The values are represented as mean ± standard deviation.

### 2.9. Statistical Analysis

Statistical analyses of the obtained data were performed using two-tailed unequal variance Student's *t*-tests, one-way ANOVA with Tukey's multiple comparisons tests, Chi-square tests, two-way ANOVA with Tukey's multiple comparisons tests, linear regression, and nonlinear regression. Significance was considered as *P* < 0.05. Mean and standard deviation or standard error of the mean (as noted in each figure legend) were plotted using GraphPad Prism V8. For traditional 2D culture experiments, *n* represents the number of independent experiments. Three to five (nonoverlapping) fields of view were analyzed for each group in each independent experiment. For engineered tissue experiments, *n* represents the number of experiments with independent hiPSC-cardiomyocyte differentiation batches. Within each of these experiments, Six to eight tissues were tested per group. The number of independent experiments is reported in each figure legend, and the number of tissues measured is reported on data bars, where applicable.

## 3. Results

### 3.1. Human Cardiac Fibroblasts Undergo Myofibroblast Activation in Monolayer Culture

**Figure 1:**
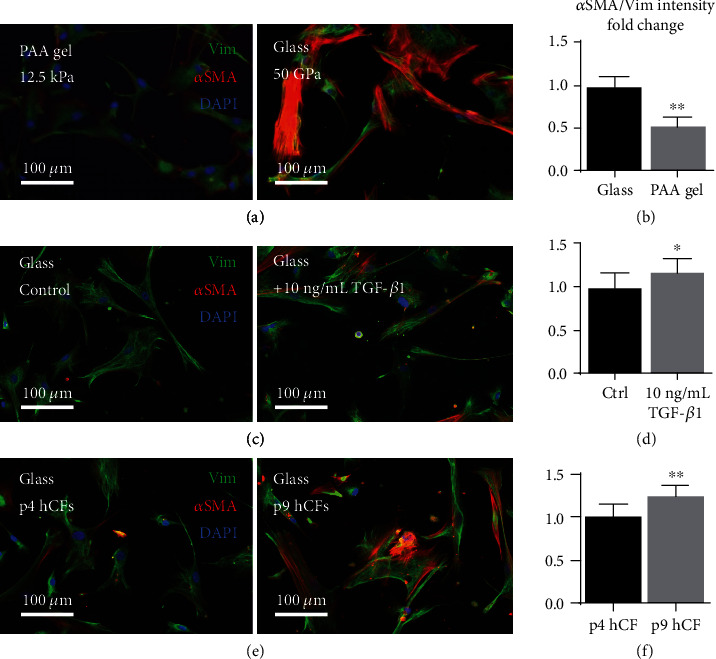
Human cardiac fibroblasts display classic signs of activation in monolayer culture. (a and b) HCFs cultured on soft 12.5 kPa polyacrylamide gels or stiff 50 GPa glass were stained for vimentin (Vim) and *α*-smooth muscle actin (*α*SMA (a)), and fluorescence intensity was quantified ((b) ^∗∗^*P* < 0.01, *n* = 5). (c and d) HCFs cultured on glass were stimulated with 10 ng/mL TGF-*β*1, stained (c), and quantified ((d) ^∗^*P* < 0.05, *n* ≥ 10). (e and f) Early-passage (p4) and late-passage (p9) human cardiac fibroblasts (^∗∗^*P* < 0.01, *n* ≥ 17). Data are represented as mean ± SD.

Because adult human cardiac fibroblasts (hCFs) are a novel cell type to include in our engineered cardiac tissues (ECTs), we sought to evaluate their phenotype in 2D culture. Sensitivity of hCFs to stiffness-induced myofibroblast activation was determined by plating passage 3 or 4 (p3 or p4) hCFs on polyacrylamide gels or glass coverslips with stiffnesses of approximately 12 kPa and 50 GPa, respectively. After 72 hours of culture, hCFs were fixed and immunofluorescently labeled for vimentin, an intermediate filament protein in fibroblasts, and alpha-smooth muscle actin (*α*SMA), an actin isoform whose increased expression is associated with myofibroblast activation (Figure 1(a)) [37]. Expression of *α*SMA normalized by vimentin was 0.5-fold less in hCFs cultured on polyacrylamide gels of a physiological stiffness compared to those cultured on glass (*P* < 0.01, *n* = 5; Figure 1(b)), demonstrating hCF sensitivity to stiffness-induced activation in 2D culture.

We next assessed the ability of hCFs to be further activated via biochemical stimulation mimicking inflammation by seeding passage 4 hCFs on glass coverslips and treating them with 10 ng/mL TGF-*β*1 for 48 hours. Cells were subsequently fixed and fluorescently labeled for *α*SMA and vimentin (Figure 1(c)). TGF-*β*1-treated hCFs had a 1.2-fold increase in normalized *α*SMA fluorescence compared to control (*P* < 0.01, *n* = 3 per group; Figure 1(d)). These data show that in addition to stiffness-induced myofibroblast activation, biochemical activation of hCFs occurs in 2D culture.

To further evaluate hCF activation in response to prolonged exposure to a stiff substrate, cells were maintained on tissue culture plastic with a stiffness of approximately 100 MPa from passage 4 (p4) to passage 9 (p9). Cells were seeded on glass coverslips prior to staining and imaging, and passage 9 hCFs showed a 1.3-fold increase in *α*SMA expression over p4 hCFs (*P* < 0.01, *n* = 3 per group; Figures 1(e) and 1(f)). mRNA was collected from p4 and p9 hCFs and p8 neonatal human dermal fibroblasts (NHDFs), and q-RT-PCR was run to assess transcript expression levels. Analysis revealed a significant increase in transcript levels of ACTA2, encoding *α*SMA, by 1.5-fold in p9 hCFs over p4 hCFs (*P* < 0.05, *n* ≥ 3; Supplement Figure S1A). Additionally, GJA1, encoding connexin 43 (Cx43), which has been shown to be present in cardiac fibroblasts [26, 38, 39] and upregulated in parallel with *α*SMA during myofibroblast activation [40], increased by 4.6-fold in p9 over p4 hCFs (*P* < 0.01, *n* ≥ 3; Figure S1A). Notably, expression levels of both ACTA2a and GJA1 were significantly lower in NHDFs than hCF groups, while levels of housekeeping gene expression (HPRT) were similar across groups. These data support the hypothesis that fibroblast lineage impacts cellular phenotype and heterotypic interactions, which becomes important for cardiac tissue formation and electromechanical function.

### 3.2. Human Cardiac Fibroblast Percentage Modulates Engineered Tissue Formation, Mechanics, and Kinetics

**Figure 2:**
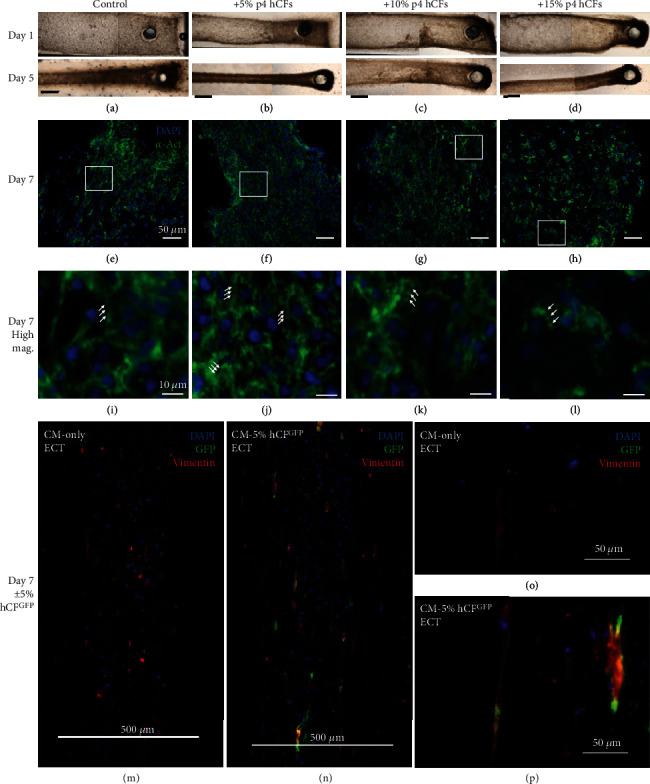
Human cardiac fibroblasts (hCFs) change tissue-level compaction and organization. (a–d) Phase contrast images of engineered cardiac tissues with no added hCFs ((a) Control), 5% p4 hCFs added (b), 10% p4 hCFs added (c), or 15% p4 hCFs added (d) were taken at one day after formation (Day 1, top row) and five days after formation (Day 5, bottom row). Scale bar is 1 mm. (e–h) Fluorescence images of control (e), 5% hCF (f), 10% hCF (g), and 15% hCF (h) tissues 7 days after formation (Day 7) stained for nuclei (DAPI, blue) and cardiomyocyte marker *α*-actinin (*α*-Act, green). Scale bar is 50 *μ*m. (i–l) Magnified portions of boxes outlined in white in e–h. White arrows indicated striations. Scale bar is 10 *μ*m. (m–p) Fluorescence images of ECTs with cardiomyocytes only (m, o) and 5% human cardiac fibroblasts labeled with GFP (hCF^GFP^ (n, p)) at low ((m, n) scale bar is 500 *μ*m) and high ((o, p) scale bar is 50 *μ*m) magnification. Tissues are stained for GFP (labeled hCFs, green) and vimentin (red).

Addition of hCFs to our 3D ECT platform enabled examination of their effect on hiPSC-cardiomyocytes in a physiologically relevant 3D tissue microenvironment. In the human heart, cardiomyocytes compose ~20-30% of cells by number [14]; however, hiPSC-cardiomyocyte volume is approximately 1/3 of the adult human cardiomyocyte volume [29]. In order to recapitulate an appropriate volume ratio of cardiomyocytes to noncardiomyocytes in ECTs, hCFs were doped in at a range of 0%-15% of input hiPSC-cardiomyocytes. In hiPSC-cardiomyocytes, hCF tissues were formed and cultured for one week. Tissues in all groups compacted the collagen matrix and formed a beating syncytium by day three of culture (Figures 2(a)–2(d)). After one week of culture, tissues from all groups contained striated cardiomyocytes throughout, where hiPSC-cardiomyocytes were most densely compacted with areas of local fiber alignment in 5% hCF-containing tissues and conversely were less dense and lacked local alignment in 15% hCF-containing tissues (Figures 2(e)–2(l)). By forming ECTs with 5% hCFs infected with lentivirus to express GFP, we confirmed uniform distribution of GFP^+^ hCFs in engineered tissues after one week of culture (Figures 2(m)–2(p)). The GFP^+^ hCFs were uniformly positive for vimentin, a fibroblast marker, and did not overtake the cellular composition of ECTs. Tissues formed with and without hCFs both contain some vimentin-positive cells, suggesting that some of the noncardiomyocytes from hiPSC differentiation have a fibroblast-like phenotype. This is supported by flow cytometry analysis of differentiated populations, where the population of *α*SMA single positive cells made up 23.3% ± 4.7% of all cells (means ± SEM).

**Figure 3:**
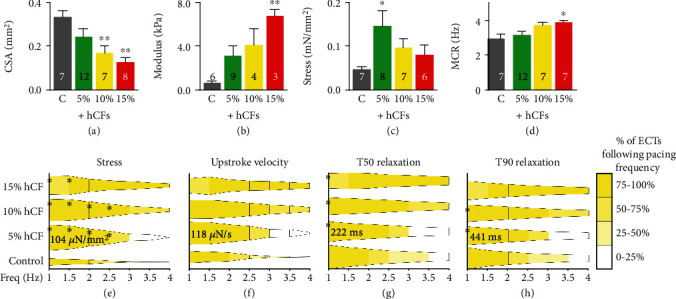
Mechanical and kinetic properties of engineered tissues are sensitive to hCF doping after one week of culture. (a) Cross-sectional area (CSA) of tissues was measured at relaxed length. (b) Young's Modulus was measured by stretching tissues from relaxed length to 130%. (c) Active stress was measured as the average peak contractile twitch force of tissues paced at 1 Hz field stimulation. (d) Maximum capture rate of tissues was measured during pacing from 1 Hz to 4 Hz field stimulation. ^∗^*P* < 0.05 and ^∗∗^*P* < 0.01. Data are represented as mean ± SEM. (e–h) Tissue force amplitude and kinetics were measured as a function of increasing pacing frequency. Active stress (e), upstroke velocity (f), time to 50% relaxation from peak stress (T50 (g)), and time to 90% relaxation from peak stress (T90 (h)) are shown normalized to 5% hCF tissues at 1 Hz (value reported on graphs). Width of bars represents relative amplitude, and color of bars represents the percentage of tissues following the pacing with 1 : 1 capture rate compared to 1 Hz. Bold numbers indicate the value of a respective metric in 5% hCF tissues at 1 Hz pacing. ^∗^*P* < 0.05 versus control at same frequency. *n* = 3 independent experiments; the number of tissues per measurement is indicated on each bar. C: cardiomyocyte-only control; 5%, 10%, and 15%: addition of 5%, 10%, or 15% human cardiac fibroblasts, respectively, with the number of cardiomyocytes held constant.

HCFs increased ECT compaction as measured by cross-sectional area in a dose-dependent manner, resulting in a 25% to 63% decrease in cross-sectional area which was a significant reduction with 10% and 15% hCFs (*P* < 0.01, *n* = 3; Figure 3(a); Table S2). Importantly, similar results could not be obtained with neonatal human dermal fibroblasts (NHDFs), which interrupted tissue formation and function (Supplement Figure S2, Supplement Movie S1). These results with dermal fibroblasts indicate that fibroblast origin is a critical factor for determining their impact on not only ECT formation but also ECT function (Supplement Figure S2).

Mechanical properties of ECTs were tested after 7 days of culture to determine the effect of ECT cellular composition on functional performance early after tissue formation before natural maturation processes progressed through longer-term culture periods. Tissues were mounted on a custom mechanical apparatus between a force transducer and a digitally controlled motor and bathed in 37°C Tyrode's solution with 1.8 mM CaCl_2_. Tissue stiffness increased with the addition of hCFs in a dose-dependent manner by 3.7-fold with 5% hCFs, 5.2-fold with 10% hCFs, and significantly by 9.1-fold with 15% hCFs (*P* < 0.01, *n* = 3; Figure 3(b); Table S2). Active stress, the contractile force produced during twitch contractions normalized by cross-sectional area, was measured at 1 Hz stimulation and increased significantly with the inclusion of 5% hCFs. However, tissues containing 10% and 15% hCFs did not differ significantly from control (*P* < 0.05, *n* = 3; Figure 3(c); Table S2). Together these results indicate that although the material properties of ECTs are dose dependent upon hCF inclusion, force output has a more nuanced response to noncardiomyocyte content.

We examined the effect of increasing hCF percentage on the contractile force and kinetics of engineered cardiac tissues during a frequency ramp protocol of 0.5 Hz increments from 1 Hz up to 4 Hz field stimulation. Because hCFs can electrically couple to cardiomyocytes via gap junctions to participate in electrical propagation in both the healthy and diseased heart [27, 41, 42], we hypothesized that their inclusion would not hinder electrical activity in ECTs. Maximum capture rate (MCR) was measured at peak tissue length and was near peak human heart rates (200 bpm or 3.3 Hz) for all conditions. MCR increased in a dose-dependent manner with increasing hCF percentage, reaching significance with 15% hCFs at 3.9 Hz, beyond that of physiological heart rates [43] (*P* < 0.05, *n* = 3; Figure 3(d); Table S2).

The force-frequency relationship in engineered cardiac tissues was determined by measuring contractile force while pacing tissues from 1 Hz to 4 Hz at 0.5 Hz steps. ECTs in all groups showed reduction in force amplitude with increasing pacing frequency, as described in our previous studies with this platform [35] and by others [44–46]. Increasing hCF content did not alter the force-frequency response, and all tissues experienced a significant decrease in contractile force between 2 and 3.5 Hz compared to 1 Hz (*P* < 0.05, *n* = 3; Figure 3(e); Table S3). Importantly, force amplitude of 5% hCF tissues was significantly greater than control ECTs between 1 and 2.5 Hz, indicating that the benefit of 5% hCFs to contractile force extends across a range of physiological frequencies (*P* < 0.05, *n* = 3; Figure 3(e); Table S3).

To determine the frequency dependence of contraction and relaxation kinetics in ECTs, we analyzed the upstroke velocity (*v*_up_, defined as the steepest slope of contraction rise), time to 50% relaxation of peak force (T50), and time to 90% relaxation of peak force (T90). *v*_up_ did not differ significantly between groups (Figure 3(f)); however, relaxation kinetics were significantly altered with the addition of 5%-15% hCFs at resting heart rates. At 1 Hz (60 bpm), T50 and T90 were significantly reduced in hCF-containing tissues compared to control, indicating faster relaxation (*P* < 0.05, *n* = 3; Figures 3(g) and 3(h); Table S3). The relationship between relaxation time and contractile force was significantly steeper in control tissues (Figures S3B and S3C); this means that for a given level of force, the addition of cardiac fibroblasts significantly reduces the relaxation time to speed relaxation.

**Figure 4:**
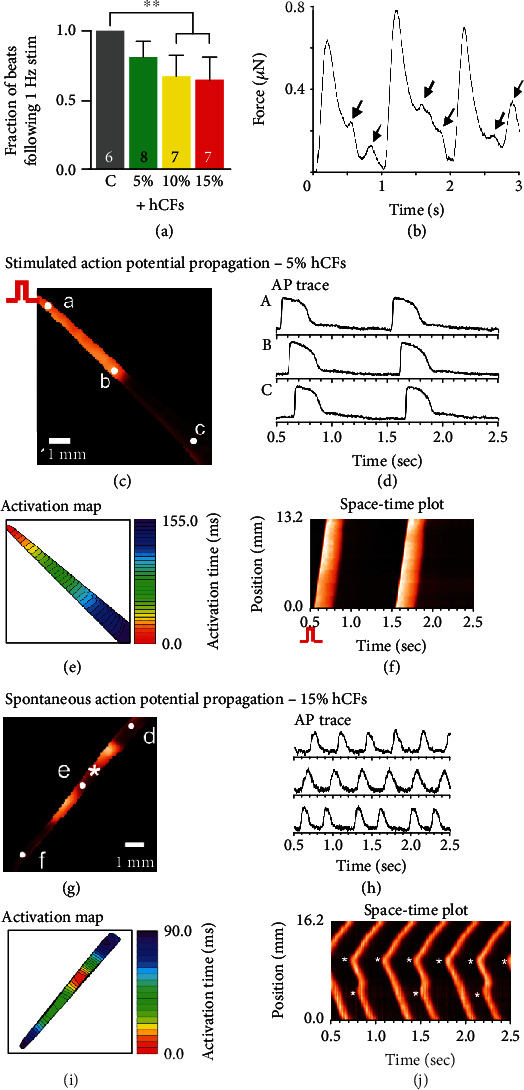
Higher percentages of human cardiac fibroblasts increase spontaneous beating activity. (a) Fraction of beats captured at 1 Hz pacing at 130% *L*_0_ (C: cardiomyocyte-only control; 5%, 10%, 15%: addition of 5%, 10%, or 15% human cardiac fibroblasts; ^∗∗^*P* < 0.01, *n* = 3 independent experiments; the number of tissues measured is indicated on each bar; data represent mean ± SEM). (b) Example raw trace of 15% hCF tissue during 1 Hz pacing. Arrows indicate triggered activity similar to premature ventricular contractions (PVCs) in the clinical setting. (c–j) Representative maps of action potential propagation of paced or spontaneous beats. (c) Fluorescence image of paced beat from 5% hCF ECT, stimulated from the left upper corner. (d) Sample action potential traces from the locations marked with “A,” “B,” and “C” in (c). (e) Action potential propagation map. (f) Space-time plot of action potential propagation (conduction velocity (CV) = 6.42 cm/s, Movie S2). (g) Fluorescence image of spontaneous beat from the 15% hCF ECT. (h) Sample action potential traces from the locations marked with “D,” “E,” and “F” in (g). Unlike ventricular-like action potentials with a prominent plateau phase followed by a rapid repolarization as seen in 5% hCF ECT (g), spontaneously beating 15% hCF ECTs showed pacemaker-like action potentials with slow upstrokes and a triangular shape. (i) Action potential propagation map. (j) Space-time plot of action potential propagation (CV = 2.59 cm/s, Movie S3). Note that the spontaneous beats originated in two places marked with white stars (∗) and propagated in both directions.

Careful examination of the raw data traces from contractile measurements demonstrated nonuniform activation, reminiscent of arrhythmogenic behavior. We then analyzed the fraction of pulses captured by tissues within each group with 1 Hz stimulation. 10% and 15% hCF-containing tissues followed a significantly lower fraction of 1 Hz stimulation compared to control (*P* < 0.01, *n* = 3; Figure 4(a)). In fact, tissues with higher hCF content tended to respond to 1 Hz stimulation but exhibit frequent premature ventricular contractions (PVCs; Figure 4(b)) with a 2 or 3 Hz spontaneous rate, lacking an ability to rest in diastole (Figure 4(b)). The significantly decreased ability of 10% and 15% hCF tissues to follow at 1 Hz makes them less desirable for translational purposes, where it is critical that engineered tissues be capable of following within the range of the average human resting heart rate (~1 Hz or 60 bpm). Additionally, optical mapping of ECTs with higher hCF concentrations frequently showed spontaneous beating frequencies greater than 1 Hz (Figures 4(c)–4(j); Movies S2 and S3). ECTs containing 5% hCFs were able to be mapped at 1 Hz stimulation and tended to exhibit ventricular-like action potentials (APs; Figures 4(c) and 4(d)) and uniform AP propagation (Figures 4(e) and 4(f)). 15% hCF tissues beat spontaneously at 2 Hz and exhibited ectopic AP generation (Figures 4(g) and 4(h)). The activation map and space-time plot of action potential propagation indicate that spontaneous beats originate from the multiple sites (white stars) and propagate slowly with nonuniform propagation (Figures 4(i) and 4(j); Movie S3), in line with PVCs observed during the contractile measurements in Figure 4(b). The results provided further evidence that excessive fibroblasts disrupt electromechanical performance and facilitate PVCs, and that 5% hCFs were an optimal concentration for functionally normal ECTs.

### 3.3. Contractile Alternans Appear in Engineered Tissues during Increased Pacing Frequency

**Figure 5:**
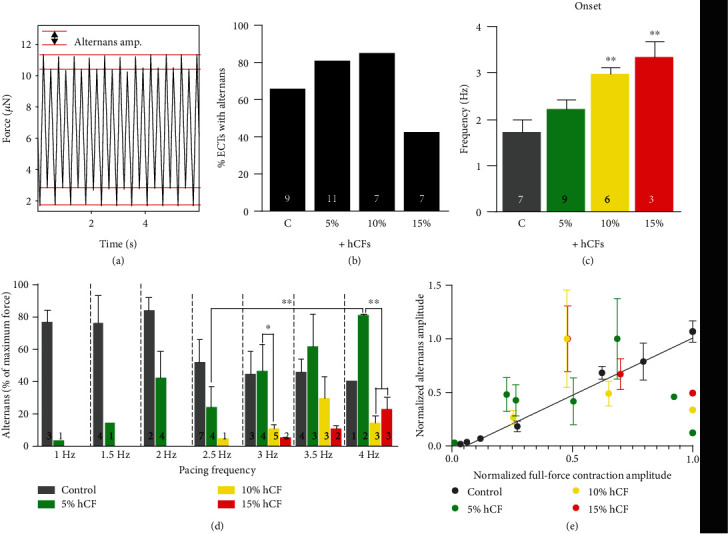
Alternans appear in engineered cardiac tissues dependent on pacing frequency and hCF percentage. (a) Raw force trace acquired during field stimulation at 2.5 Hz. Dashed lines indicate the difference in force amplitude between alternating contractions in systolic alternans (top) and diastolic alternans (bottom). (b) Percentage of tissues exhibiting alternans. (c) The onset of alternans was measured as the pacing frequency at which alternans amplitude reached ≥5% of the total force amplitude. (d) Amplitude of alternans was measured during field stimulation of increasing frequency, and the fraction of full-force contractions was calculated. (e) Normalized alternans amplitude was plotted as a function of the normalized full-force contractile amplitude. The linear regression for control tissues is shown (*R*^2^ = 0.86). *n* = 3 independent experiments; the number of tissues per measurement is indicated on each bar. C: control; 5%: +5% hCFs; 10%: +10% hCFs; 15: +15% hCFs. ^∗^*P* < 0.05 and ^∗∗^*P* < 0.01; data are represented as mean ± SEM.

In order to further elucidate how human cardiac fibroblasts affected ECT frequency-dependent behavior, we analyzed the appearance and amplitude of contractile alternans, a condition where the heartbeat cyclically varies between weak and strong (Figure 5(a)). Alternans are highly correlated with cardiac arrhythmia and have complex mechanisms [47] but have been difficult to study in human, thus their appearance in our ECTs presented a valuable opportunity for their characterization. The fraction of ECTs with alternans present was not significantly different between groups (Figure 5(b)); however, the pacing frequency necessary to elicit alternans was significantly higher in 10% and 15% hCF tissues compared to control (*P* < 0.01, *n* = 3; Figure 5(c)). We then analyzed the frequency dependence of the amplitude of alternans (Figure 5(d)).

Because the average maximum capture rate of control and 5% hCF tissues was below the pacing frequency of the average onset of alternans in 10% and 15% hCF groups, it was difficult to compare alternans behavior across the tested frequency range. To compare the relationship between alternans amplitude and full-force contraction amplitude between groups, we plotted their normalized values (Figure 5(e)). Only control tissues exhibited a significant, positive relationship between alternans and full-force contractile amplitude (*R*^2^ = 0.86). This may reflect that at low frequencies, where force production is greatest, control tissues exhibit a significant alternans amplitude, whereas hCF-containing tissues suppress alternans at these physiologically lower pacing frequencies (i.e., higher force production). Notably, high alternans amplitude at low frequencies occurred in control tissues of one biological replicate (i.e., hiPSC-cardiomyocyte differentiation run). This supports the hypothesis that the addition of hCFs reduces functional variability resulting from variable differentiation outcomes.

### 3.4. Cardiac Fibroblast Activation Reduces Mechanical Benefits of 5% hCF Addition

**Figure 6:**
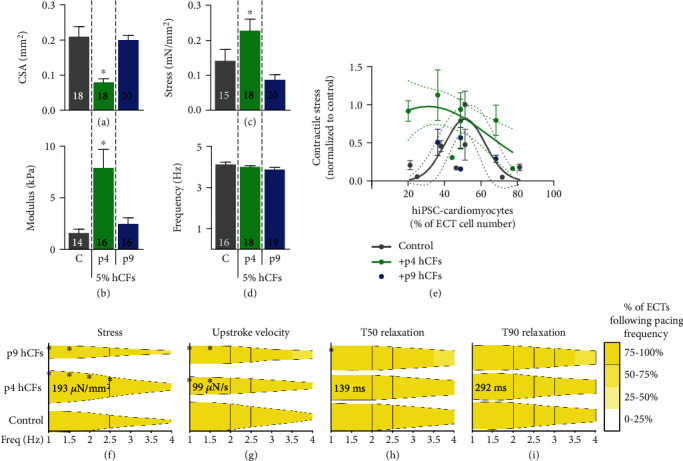
P9 hCFs reduce mechanical performance but maintain kinetic properties imparted by p4 hCFs after one week of culture. (a) Cross-sectional area (CSA) of tissues was measured at relaxed length. (b) Young's Modulus was measured by stretching tissues from relaxed length to 130%. (c) Active stress was measured as the average peak contractile force of tissues paced at 1 Hz field stimulation. (d) Maximum capture rate of tissues was measured during pacing from 1 Hz to 4 Hz field stimulation. *n* = 4 independent experiments, number of tissues tested is indicated on each bar, ^∗^*P* < 0.05, and data are represented as mean ± SEM. (e) Contractile stress normalized to maximum stress of control (hiPSC-cardiomyocyte-only) tissues as a function of engineered cardiac tissue (ECT) cardiomyocyte purity. Gaussian curves fitted to control and +p4 hCFs with dotted lines indicating 95% confidence interval. Each point represents results from an independent experiment (unique cardiomyocyte purity), with 2-6 tissues measured per group per experiment; data are represented as mean ± SEM. (f–i) Tissue force amplitude and kinetics were measured as a function of increasing pacing frequency. Active stress (f), upstroke velocity (g), time to 50% relaxation from peak stress (T50 (h)), and time to 90% relaxation from peak stress (T90 (i)) are shown normalized to the value of a given experimental condition at 1 Hz. Width of bars represents relative amplitude, and color of bars represents the percentage of tissues following the pacing with 1 : 1 capture rate compared to 1 Hz. Bold numbers indicate the value of a respective metric in 5% p4 hCF tissues at 1 Hz pacing. *n* = 4 independent experiments.

Because 5% human cardiac fibroblasts (hCFs) resulted in tissues with the highest contractile function and minimized arrhythmogenic phenotypes, we proceeded with studies at this hCF concentration. To determine the sensitivity of our tissues to cardiac fibroblast activation state, we compared the effect of 5% early-passage (p4) hCFs and 5% serially passaged (p9) hCFs to control, non-hCF-containing tissues. Taking into account the results of our experiments in traditional 2D culture, we hypothesized that the increased myofibroblast-like activation state of p9 hCFs would negatively impact the function of ECTs in which they were included, determined by metrics of stiffness, compaction, contractile force, and maximum capture rate (MCR). Tissues were formed and cultured in the same manner described above and underwent mechanical testing after one week of culture. p4 hCFs significantly increased compaction, tissue stiffness, and contractile force, with no significant difference on MCR, matching the results seen in the earlier doping experiments (*P* < 0.05, *n* = 4; Figures 6(a)–6(d); Table S4). p9 hCFs, however, showed no significant difference from control, failing to provide the same positive impact as early-passage hCFs.

Maximum active stress of tissues was normalized to control (non-hCF-containing tissues) and compared across a range of hiPSC-cardiomyocyte purities. Purity was determined by obtaining a sample of hiPSC-cardiomyocytes directly prior to tissue formation and measuring the percentage expressing cardiac troponin T (cTnT) by flow cytometry. Force production of control tissues exhibited a bell-curve response to purity with maximum force at 51% ± 11% hiPSC-cardiomyocyte purity (*R*^2^ = 0.56; Figure 6(e)), similar to previously reported results [11]. The addition of 5% p4 hCFs (but not p9 hCFs) extended the range of hiPSC-cardiomyocyte purity resulting in higher force production to include lower percentages of cardiomyocytes. These results indicate that a low percentage of early-passage cardiac fibroblasts is a valuable tool for not only maximizing force but also preserving that force over a wider range of input hiPSC-cardiomyocyte purities.

### 3.5. Activated Cardiac Fibroblasts Modulate Contraction and Relaxation Kinetics

The frequency dependence of force production, contraction velocity, and relaxation times was measured with the addition of 5% p4 or p9 hCFs. As with previous experiments, p4 hCF tissues produced significantly more force (by 1.6-fold) over control tissues between 1 and 2.5 Hz. The addition of p9 hCFs, however, resulted in a significant decrease in force compared to control and p4 hCF tissues at low pacing frequencies (*P* < 0.05, *n* = 4; Figure 6(f); Table S5). This suggests that not only do p9 hCFs abrogate the benefits of 5% hCFs, but they in fact inhibit or reduce contractile function of ECTs.

Similar to our hCF doping experiments, upstroke velocity increased faster with increasing force in control tissues compared to hCF-containing ECTs (*P* < 0.05, *n* = 4; Figures 6(g) and S5A; Table S5). Relaxation times were similar across all groups compared at the same pacing frequencies (Figures 6(h) and 6(i)); however, the force dependence of relaxation kinetics differed significantly. The relationship between relaxation times and contractile stress was fitted with linear regressions for T50 and T90, and p9 hCF ECTs had significantly larger slopes for both relaxation time parameters compared to control and p4 hCF tissues (*P* < 0.01, *n* = 4; Figure S5). For a given increase in contractile stress, p9 tissues displayed a significantly larger increase in relaxation time, reminiscent of increased relaxation times in cardiac infarct scar tissue.

### 3.6. Contractile Alternans Reflected in Ca^2+^ Transient Alternans

**Figure 7:**
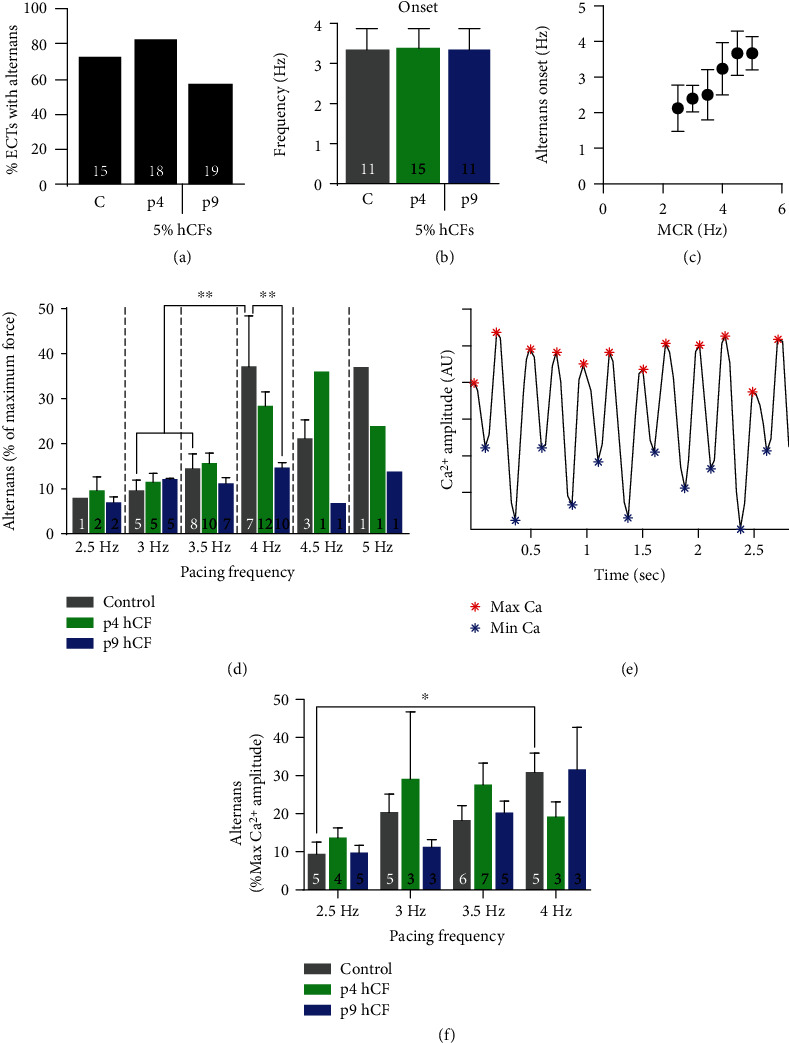
Contractile alternans appear independently of early or late-passage hCFs but have a higher force threshold in early-passage tissues. (a) Percent of tissues with alternans. (b) The onset of alternans was measured as the pacing frequency at which alternans amplitude reached ≥5% of the total force amplitude. (c) Pacing frequency of alternans onset plotted as a function of maximum capture rate (MCR; *n* = 7 for (c) only). (d) Amplitude of alternans was measured during field stimulation of increasing frequency and represented as percentage of full-force contractile amplitude. (e) Example raw trace of Ca^2+^ amplitude by Rhod-2 fluorescence during 2 Hz stimulation. Red and blue markers denote local maxima and minima, respectively. (f) Alternans in Ca^2+^ amplitude as a function of pacing frequency. *n* = 4 independent experiments; the number of tissues per measurement is indicated on respective bars. C: control cardiomyocyte-only tissues; p4: 5% p4 hCF-added tissues; p9: 5% p9 hCF-added tissues. ^∗^*P* < 0.05 and ^∗∗^*P* < 0.01; data are represented as mean ± SEM.

In order to determine how cardiac fibroblast activation state affected alternans behavior in ECTs, we analyzed the fraction, onset, and amplitude of alternans during pacing from 1 Hz to 5 Hz. The fraction of tissues exhibiting alternans did not differ significantly between groups and was similar in control and 5% p4 hCF ECTs to results obtained in hCF doping experiments (*n* = 4; Figure 7(a)). Onset of alternans did not significantly differ and occurred between 3 and 4 Hz pacing in all groups (*n* = 4; Figure 7(b)). The onset of alternans in control and 5% p4 hCF ECTs was significantly higher in these experiments compared to their counterparts in hCF doping experiments (*n* ≥ 3, *P* < 0.01; Figures 5(c) and 7(b)) which is likely due to the significantly faster maximum capture rate in these tissues (*n* ≥ 3, *P* < 0.01; Figure 7(c)). Control tissues had a significant increase in alternans amplitude at 4 Hz compared to 3 and 3.5 Hz (*P* < 0.01, *n* = 4; Figure 7(d)); however, the addition of both 5% p4 or p9 hCFs removed frequency dependence of alternans amplitude. These results, taken together with those of hCF 5-15% doping experiments, suggest that alternans onset is related to a tissue's maximum capture rate and that the addition of cardiac fibroblasts dampens the frequency dependence of alternans amplitude.

To verify the presence of alternans, we performed simultaneous force and Ca^2+^ measurements using Rhod-2 Ca^2+^-sensitive dye and video recording of the fluorescence signal while stimulating tissues at increasing frequencies [48]. Alternans in contractile amplitude was verified by alternating Ca^2+^ transient amplitudes in all groups. In control tissues, Ca^2+^ alternans amplitude increased significantly with increasing frequency, reminiscent of contractile alternans results (*P* < 0.05, *n* = 3; Figures 7(e) and 7(f)). Taken together, this data demonstrates that ECTs of all groups exhibit alternans and that these alternans are present at the calcium-handling level.

## 4. Discussion

Electromechanical immaturity of stem cell-derived cardiomyocytes is a significant limitation in the advancement of cardiac tissue engineering toward translational applications, and stromal cells from various sources are gaining scientific favor for the array of benefits they provide to engineered tissues. This study describes the functional effects of human cardiac fibroblasts (hCFs) on hiPSC-cardiomyocyte engineered tissue electromechanical function, as they are sourced from the heart and have been shown to modulate both excitation and contraction [49]. We demonstrate that careful tuning of the hCF percentage and limiting the activation state by using low-passage hCFs are effective approaches to engineer physiologically healthy human myocardium *in vitro*. Novel findings of this research include (1) evidence of primary adult human cardiac fibroblast activation in 2D culture through mechanical, biochemical, and serial passaging methods; (2) identification of 5% hCF content in engineered cardiac tissues for optimal functional performance; (3) demonstration that higher hCF content significantly increases spontaneous beating frequency and leads to a smaller fraction of engineered tissues capable of following physiological pacing; (4) evidence that functional benefits imparted by early-passage hCFs are not present when late-passage (and activated) hCFs are used in 3D tissues; and (5) the first report to our knowledge of contractile and calcium alternans in human engineered cardiac tissues as a novel metric to assess tissue electromechanical maturity.

Because primary human cardiac fibroblasts have only recently been adopted as support cells for human cardiac tissues and it is well known that culture conditions can modulate cellular phenotype [50, 51], it is necessary to evaluate hCF behavior in traditional, two-dimensional culture. We used previously studied techniques to induce myofibroblast-like activation and deactivation. Transition from a soft, polyacrylamide gel to stiff, tissue culture plastic has been shown to promote stiffness-induced myofibroblast differentiation in human lung fibroblasts [50], a reversible process [51], and one we were able to replicate with hCFs. To recapitulate biochemical myofibroblast activation in hCFs, we used TGF-*β*1 [52], a growth factor demonstrated to differentiate human adipose-derived stem cells to myofibroblasts as evidenced by increased *α*SMA expression after TGF-*β*1 stimulation [53], results we were able to replicate with hCFs. Finally, we sought to validate increased myofibroblast activation with serial passaging, a phenomena which has been reported in rodent cardiac fibroblasts [54]. Through long-term culture hCFs, we were able to achieve a significant increase in *α*SMA expression and define states of low and high activation for use in engineered tissues. It is generally accepted that isolated fibroblasts obtain some level of activation when grown on stiff substrates (such as tissue culture plastic) [50], and it is thus important to consider that the *levels* of activation are graded and variable rather than producing a complete switch in phenotype from “off” to “on.” The extent to which myofibroblast activation gained in monolayer culture affects engineered tissue mechano- and electrophysiology was explored in this study, but there is more research to be done before these and other support cell types reach clinical translation.

Cardiomyocytes make up approximately 70 to 80% of the volumetric fraction of the heart [55, 56], and we have previously shown that the volume of newly differentiated cardiomyocytes is roughly one third that of neonatal cardiomyocytes [29]. In order to maintain the physiological volume ratio in our engineered tissues, we chose to dope in hCFs at a range of 5-15% of hiPSC-cardiomyocyte number. Tissues with 5% hCFs produced significantly higher contractile forces than any other group, and we used this ratio for subsequent cardiac tissue engineering experiments. Although 10% and 15% hCFs increased tissue compaction, stiffness, and maximum capture rate more significantly than 5% hCFs, the prolongation of their relaxation time and increasing frequencies and appearance of PVCs, made these higher percentages less desirable for engineering a healthy cardiac tissue. Additionally, the fraction of beats captured at 1 Hz stimulation was significantly decreased in 10% and 15% tissues compared to control, which suggest that this higher percentage of fibroblasts disrupts the electromechanical behavior of the tissues. Notably, we found that neonatal human dermal fibroblasts (NHDFs) in the same concentration range disrupted ECT formation and function, demonstrating that fibroblast origin uniquely determines fibroblast behavior. This finding is in agreement with previous reports that fibroblasts originating from different organs possess unique transcription profiles which can be distinctly grouped to dermal and nondermal fibroblasts [57].

Engineered cardiac tissues were sensitive to hCF concentration across multiple metrics, indicating the importance of heterotypic interactions between hiPSC-cardiomyocytes and hCFs. Tissue compaction, stiffness, and maximum capture rate (MCR) responded to increasing hCF concentration in a dose-dependent manner. Increased compaction and stiffness were expected, as cardiac fibroblasts are a remodeling and ECM-producing cell type; however, the increased MCR was surprising, given the previous reports that cardiac fibroblasts are nonexcitable cells which decrease beating rate with increasing percentage [58]. A possible explanation is that the higher fibroblast content may have caused cardiac myocyte electromechanical remodeling including shortening of APDs and hyperpolarizing resting membrane potentials [59], resulting in increased MCR and spontaneous activity of ECTs with 10% and 15% hCFs. Further evidence for this was demonstrated through optical mapping of ectopic activity in 15% hCF ECTs.

In order to determine the sensitivity of our ECTs to the activation state of hCFs, we compared their performance with 5% early-passage (p4) hCFs to those with 5% late-passage (p9) hCFs. p9 hCFs abrogated the benefits p4 hCFs provided in ECT compaction, stiffness, and force production after one week of culture, indicating that serial passaging leading to increased myofibroblast activation was at least partially retained during that time in three-dimensional culture. Previous work has shown that myofibroblast deactivation, in the form of decreased *α*SMA expression, occurs within 24 to 48 hours when p1 [60], p2 [61], or p2-p7 [62] fibroblasts are transferred from stiff, two-dimensional substrates to three-dimensional culture platforms. The results presented in this study suggest that the activation of p9 hCF 2D culture affects hiPSC-cardiomyocyte function in 3D engineered tissues out to at least one week. This provides an exciting avenue for engineering human myocardium of different pathophysiologies based solely on the preconditioning of hCFs.

Upon careful analysis, we discovered that some ECTs display contractile alternans (Figure 5(b)) whose amplitude increased with increasing frequency (Figure 5(d)). Cardiac alternans have been well documented clinically and are considered an indicator of arrhythmogenic risk [47, 63]. Potential involvement of CFs in modulating cardiac alternans, therefore, has been of great interest to develop effective antiarrhythmic therapy [64–66]. Cardiac fibroblasts can alter the dynamics of alternans, but the mechanisms are complex. Paracrine effect from CFs can influence alternans dynamics through causing electrophysiological remodeling of cardiomyocytes [67]. Electrical coupling of CFs to cardiomyocytes through gap junctions may accentuate the buffering effects of CFs on myocyte ion fluxes. Interestingly, p9 hCF shows increased GJA1 expression, which mirrors with gap junction remodeling in activated CFs from infarcted hearts [68]. Coupling between CF and cardiomyocytes can either promote or suppress cardiac alternans, depending on two competing effects on repolarization and Ca^2+^ cycling [69]. Further, cardiomyocytes with a relatively immature set of calcium-handling proteins, as is likely found in hiPSC-CMs [70], may be more prone to alternans due to an inability to effectively regulate calcium current (*I*_Ca_), which is a major driver of alternans [71]. In our data set, the pacing frequency at which alternans onset occurs increases with dose-dependent hCF inclusion (Figure 5(c)), likely due to the higher maximum capture rates of higher percentage hCF tissues. This higher maximum capture rate in 10% and 15% hCFs was also associated with faster spontaneous excitation rates and a pathologic inability to follow a “resting” heart rate of 1 Hz (Figure 4). The faster intrinsic rates may condition the calcium handling in order to delay the onset of alternans, but the inability to override the intrinsic rate with a slower pulse may lead to other arrhythmias. Alternans onset is well known to appear at shorter cycle lengths (higher frequencies), but our data suggests that the appearance of alternans in engineered cardiac tissue at lower beating rates may indicate an immature electromechanical phenotype. Thus, the metrics described herein to quantify alternans onset and amplitude may be useful for assessing electromechanical maturity of engineered cardiac tissues from hiPSCs.

## 5. Conclusions

In summary, this study demonstrates the functional impact of human cardiac fibroblasts on hiPSC-derived cardiomyocytes in engineered cardiac tissue. The addition of 5% resulted in an optimized, highly contractile engineered tissue. Further, we showed that increasing percentages of hCFs led to higher spontaneous beating rates and increased maximum capture rate, demonstrating their integral role in hiPSC-cardiomyocyte electrical behavior and whole-myocardium function. We showed that the functional benefits imparted by 5% hCFs were lost when hCFs were activated through serial passaging, which caused a disease-like state. Finally, we report for the first time the presence of alternans in hiPSC-cardiomyocyte engineered tissues, which is invaluable information as these tissue engineering approaches advance toward clinical application. Taken together, these results demonstrate the utility of human cardiac fibroblasts for manipulating hiPSC-cardiomyocyte function to produce physiological and pathophysiological myocardial phenotypes in vitro.

## Data Availability

The data that support the findings of this study are available from the corresponding author, KLKC, upon reasonable request.

## Supplementary Materials

Supplementary 1 Supplemental Data. Additional data gathered and analyzed during the performance of these studies.

Supplementary 2 Movie S1. 20% neonatal human dermal fibroblast engineered cardiac tissue segregates into beating and nonbeating portions at 5 days of culture.

Supplementary 3 Movie S2. Example video of stimulated action potential propagation in 5% human cardiac fibroblast engineered cardiac tissue.

Supplementary 4 Movie S3. Example video of spontaneous action potential propagation in 15% human cardiac fibroblast engineered cardiac tissue.
