# Supplementary material for: Human Cardiac Fibroblast Number and Activation State Modulate Electromechanical Function of hiPSC-Cardiomyocytes in Engineered Myocardium
Source: Stem Cells Int. 2020 Jul 16;2020:9363809. doi: 10.1155/2020/9363809 (PMC7381987; doi:10.1155/2020/9363809)
Supplement: Supplementary 1 — Supplemental Data. Additional data gathered and analyzed during the performance of these studies. [file 9363809.f1.pdf]

Human cardiac fibroblast number and activation state modulate electromechanical function of hiPSC-cardiomyocytes in engineered myocardium

Cassady E. Rupert[1], Tae Yun Kim[2], Bum-Rak Choi[2], Kareen L.K. Coulombe[1]

[1]Center for Biomedical Engineering, School of Engineering and Division of Biology and Medicine, Brown University, Providence, RI, USA

[2]Cardiovascular Research Center, Rhode Island Hospital and Alpert Medical School of Brown University, Providence, RI, USA

Supplemental Data

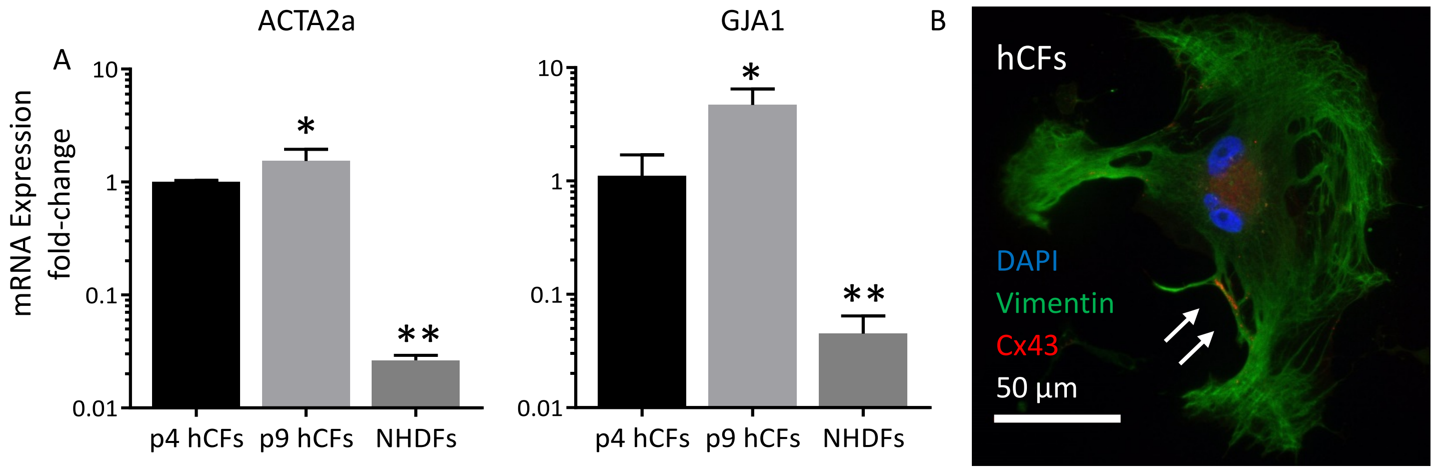

Figure S1. Myofibroblast markers upregulated in serially passaged human cardiac fibroblasts (hCFs). (A) MRNA collected from monolayer hCFs at passage 4 (p4), passage 9 (p9), or from passage 8 neonatal human dermal fibroblasts (NHDFs) was analyzed with q-RT-PCR for expression levels of ACTA2a (left, encoding α-smooth muscle actin) and GJA1 (right, encoding connexin 43). Transcript levels were internally normalized for each sample to HPRT. Data are presented as a fold-change of p4 hCF expression levels and are represented as mean ± SD, $*P<0.05$, $**P<0.01$, $n \geq 3$. (B) Passage 9 human cardiac fibroblasts (hCFs) have localized gap junctions. HCFs are labeled for vimentin (green) to identify fibroblasts, connexin 43 (Cx43, red) to identify gap junctions, and DAPI (blue) to label nuclei. Cells express both diffuse Cx43 in a peri-nuclear region (e.g., Golgi) and punctate Cx43 gap junctions at cell edges (white arrows).

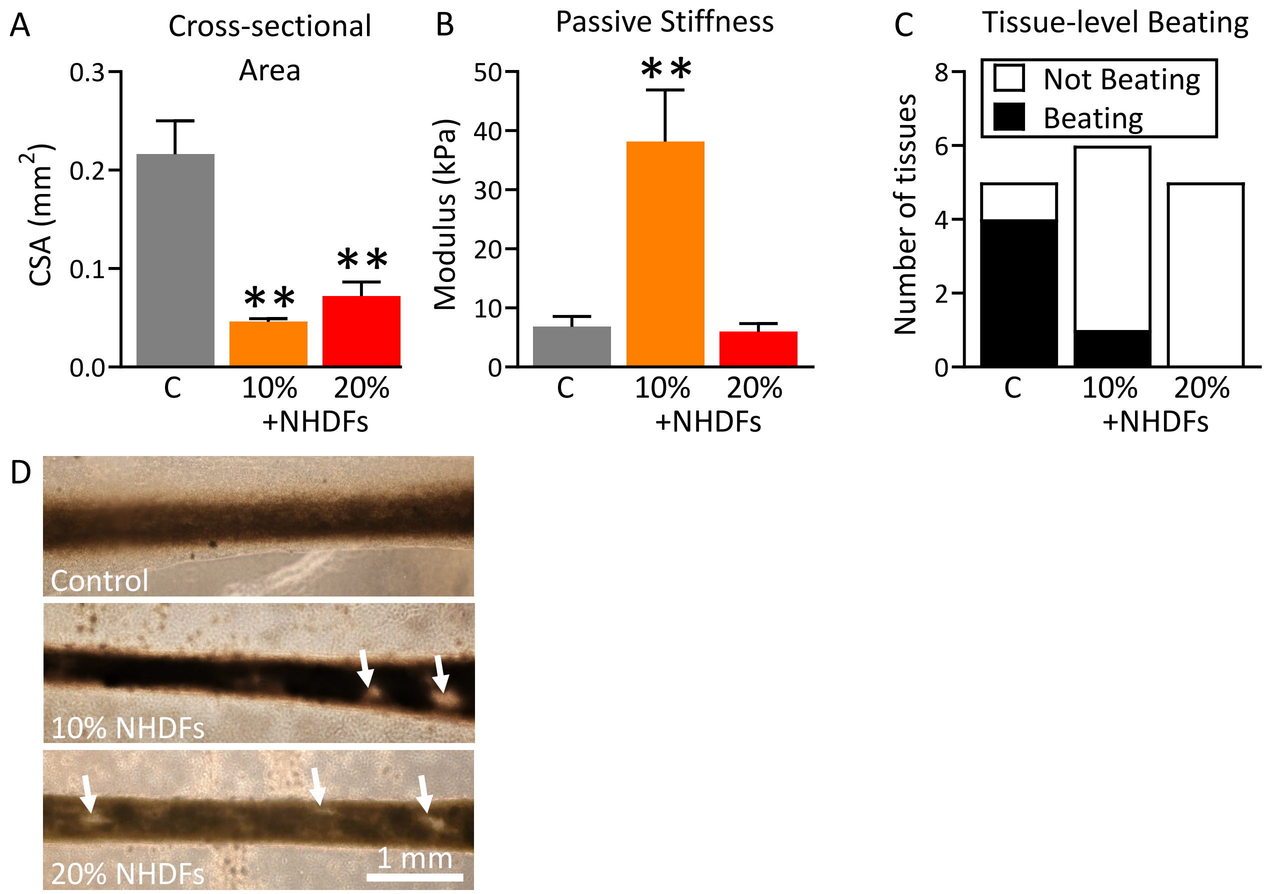

Figure S2. Neonatal Human Dermal Fibroblasts disrupt engineered cardiac tissue function. HiPSC-derived cardiomyocytes only (C, Control) or doped with 10% neonatal human dermal fibroblasts (NHDFs) or 20% NHDFs were cultured for one week. (A-B) Cross-sectional area (CSA, A) and passive stiffness (B) were measured after one week of culture. **P<0.01 by one-way ANOVA, n≥5 tissues per group, data are represented as mean ± SEM. (C) Fraction of tissues beating (black) or not beating (white) in syncytium with 1 Hz field stimulation. (D) Phase contrast micrographs of control (top), 10% NHDF-added (middle), and 20% NHDF-added tissues at 5 days of culture. White arrows indicate holes in tissues, scale bar is 1 mm.

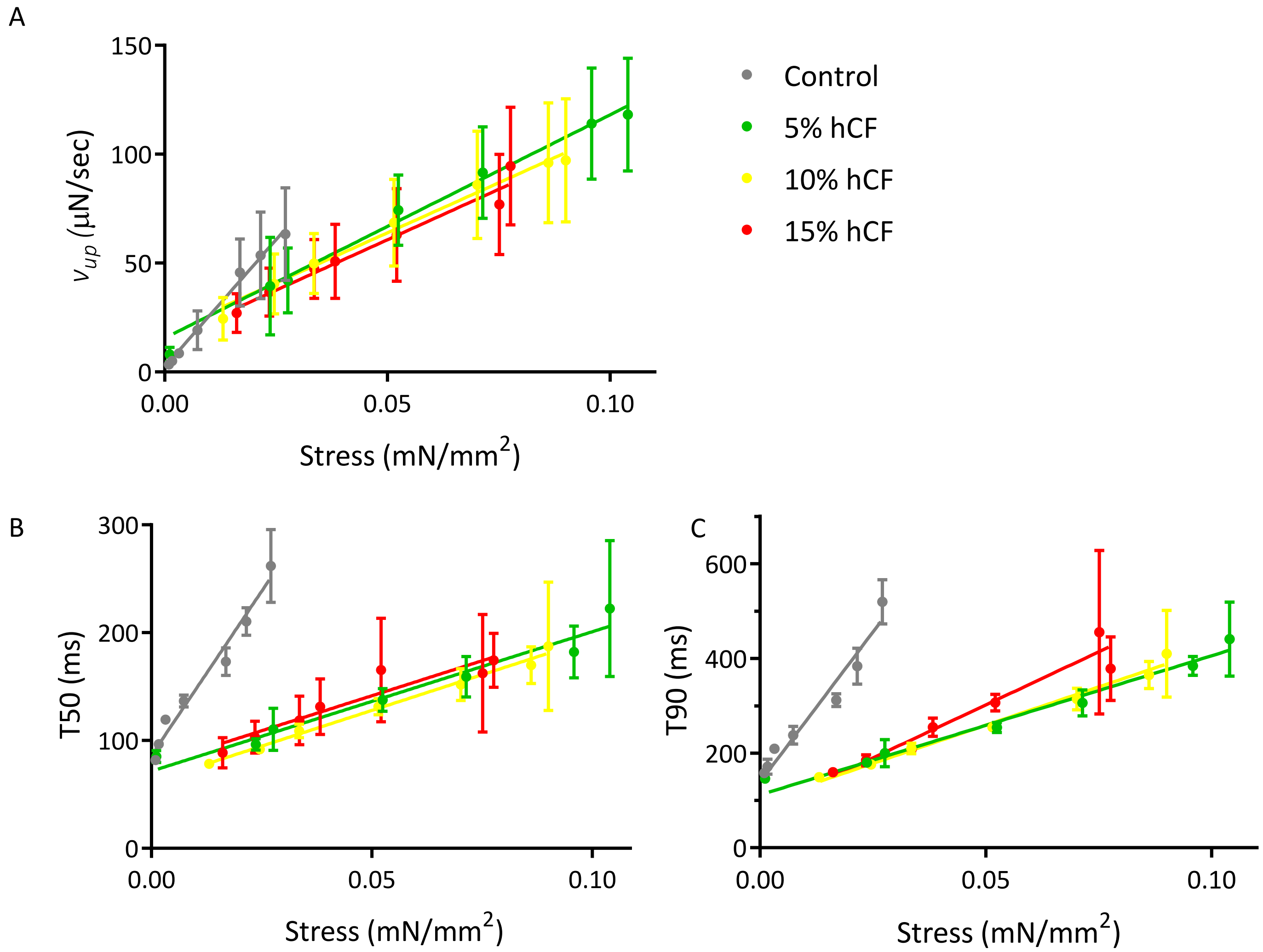

Figure S3. Contraction and relaxation kinetics of engineered cardiac tissues change with human cardiac fibroblast (hCF) addition. (A) Upstroke velocity in cardiomyocyte-only ECTs (Control), 5% hCF added ECTs (5% hCF), 10% hCF added ECTs, or 15% hCF added ECTs plotted as a function of contractile stress amplitude. (B-C) Time to 50% relaxation from peak contractile amplitude (T50; B) and time to 90% relaxation (T90; C) plotted as a function of contractile stress. The slope of the linear regression was significantly greater in control tissues compared to p4 and p9 hCF tissues. $P<0.05$, $n\geq7$, data shown as mean ± SEM (A) or SD (B-C) for ease of viewing.

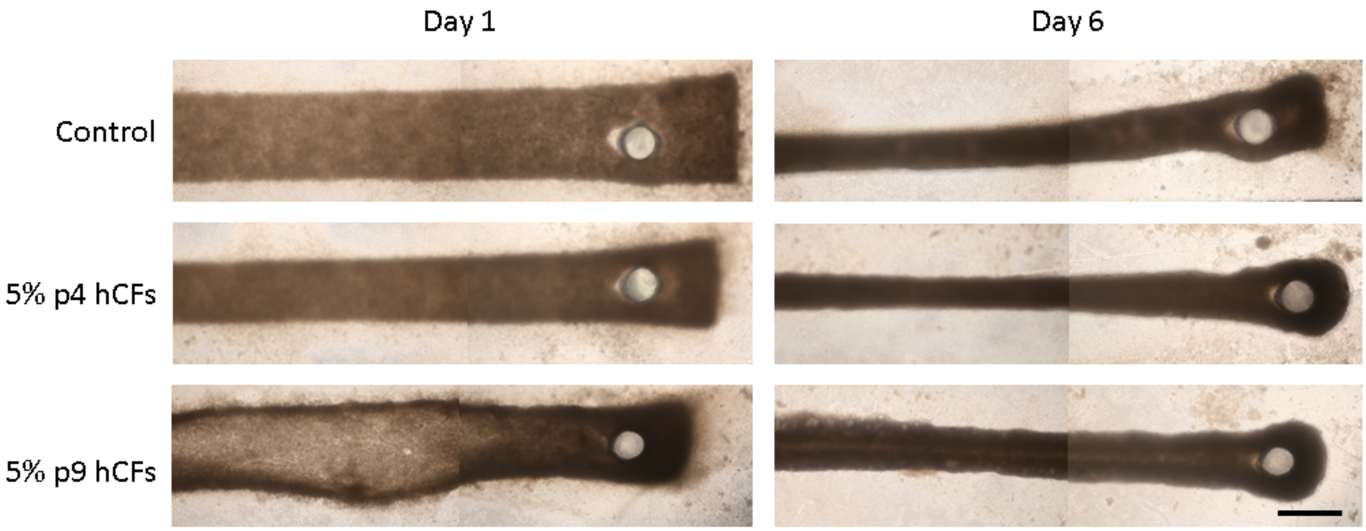

Figure S4. Engineered cardiac tissues form in the presence of early and late passage hCFs. Phase contrast images were taken at one day (Day 1, left column) and six days (Day 6, right column) after tissue formation. Tissues without hCFs (Control, top row), with 5% passage 4 hCFs (middle row), and with 5% passage 9 hCFs (bottom row) all compacted to form tissues beating in syncytia at one week. Scale bar is 1mm.

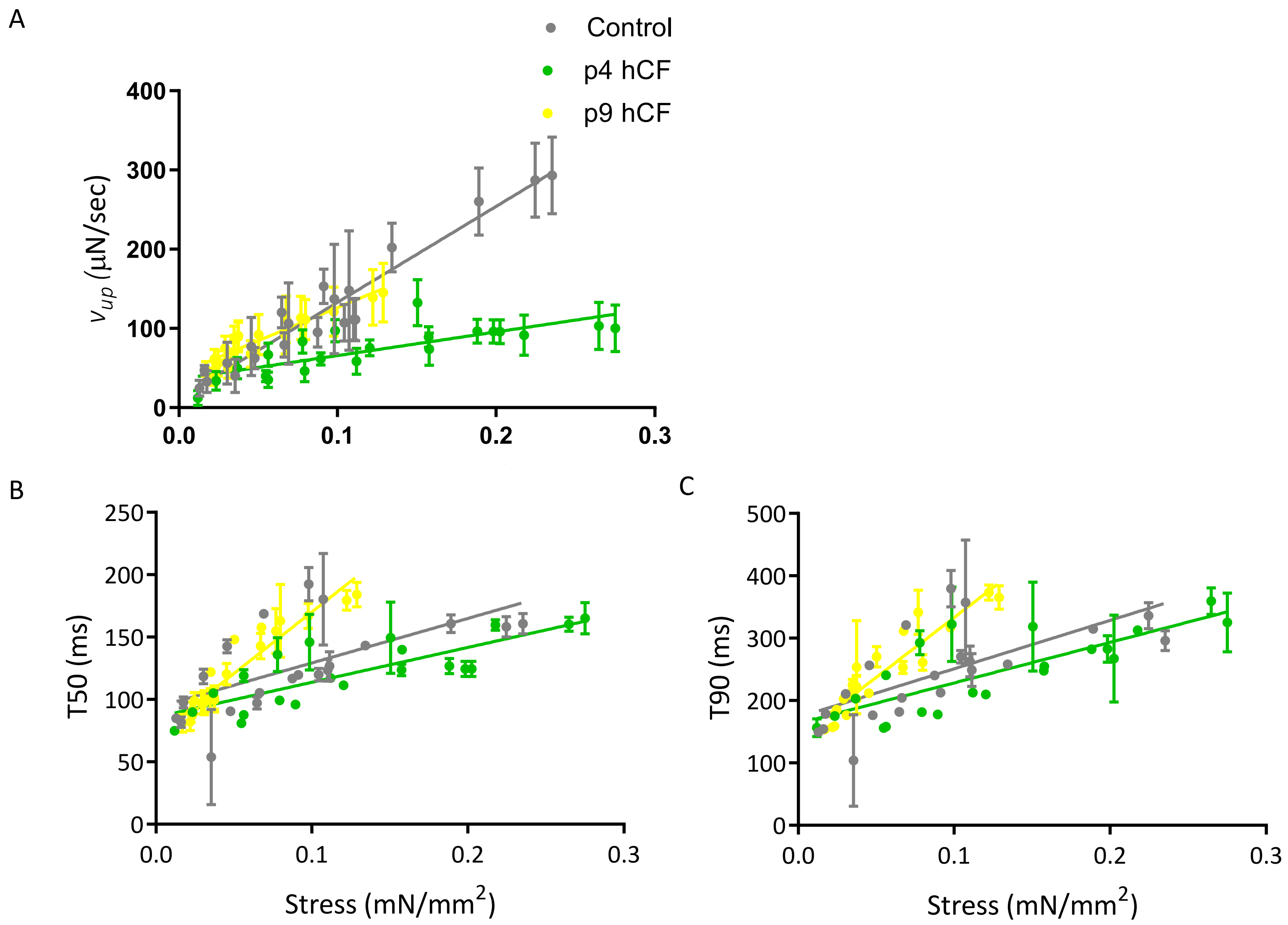

Figure S5. Contraction and relaxation kinetics of engineered cardiac tissues sensitive human cardiac fibroblast (hCF) level of activation. (A) Upstroke velocity in cardiomyocyte-only ECTs (Control), 5% p4 hCF ECTs (p4 hCF), 5% p9 hCF added ECTs (p9 hCF) plotted as a function of contractile stress amplitude. The slope of the linear regression was significantly different between all groups. (B-C) Time to 50% relaxation from peak contractile amplitude (T50; B) and time to 90% relaxation (T90; C) plotted as a function of contractile stress. The slope of the linear regression of p9 hCF tissues was significantly greater than control and p4 hCF tissues for both T50 and T90. $P<0.05$, $n\geq16$, data shown as mean ± SEM (A) or SD (B-C) for ease of viewing.

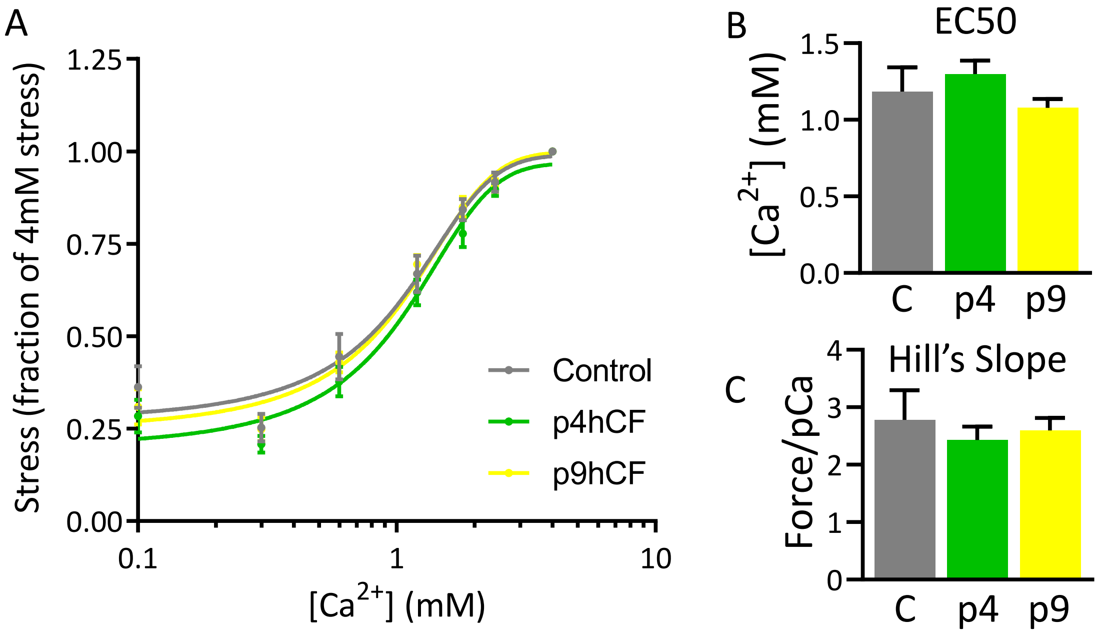

Figure S6. Human cardiac fibroblasts do not disrupt intact engineered tissues' sensitivity to extracellular Ca^{2+} concentration. (A) Intact engineered tissues were exposed to 0.1 mM to 4mM Ca^{2+} under 1 Hz pacing and contractile stress was measured. (B) EC_{50} was calculated as the extracellular Ca^{2+} concentration neces sary to reach 1.5 times baseline force. (C) Hill coefficient was calculated from the linear portion of the force-pCa curve. N≥3; data are represented as mean ± SEM.

Table S1. Custom primers used for real-time quantitative PCR.

Target	Sequence
HPRT (Hypoxanthine-guanine phosphoribosyltransferase)	Forward: TGACACTGGCAAAACAATGCA
	Reverse: GGTCCTTTTCACCAGCAAGCT
ACTA2a (α-smooth muscle actin)	Forward: CCGACCGAATGCAGAAGGA
	Reverse: ACAGAGTATTTGCGCTCCGAA
GJA1 (connexin 43)	Forward: CTTTTGGAGTGACCAGCAAC
	Reverse: TGAAGCTGAACATGACCGTA

Table S2. hCF Doping Experiments 1 Hz Mechanics Summary.

Measure	Group		Mean (n)	SEM
**Cross-sectional area (mm^2)**	Control		0.335 (7)	0.030
	5%	+ hCFs	0.243 (12)	0.036
	10%	+ hCFs	0.170 (7)	0.032
	15%	+ hCFs	0.126 (8)	0.021
**Young's Modulus (kPa)**	Control		0.67 (6)	0.11
	5%	+ hCFs	3.12 (6)	0.92
	10%	+ hCFs	4.16 (4)	1.45
	15%	+ hCFs	6.75 (3)	0.62
**Maximum active stress (mN/mm^2)**	Control		0.046 (7)	0.008
	5%	+ hCFs	0.123 (10)	0.032
	10%	+ hCFs	0.097 (7)	0.021
	15%	+ hCFs	0.080 (6)	0.024
**Maximum capture rate (Hz)**	Control		2.93 (7)	0.28
	5%	+ hCFs	3.17 (12)	0.17
	10%	+ hCFs	3.71 (7)	0.15
	15%	+ hCFs	3.86 (7)	0.14

Significant vs. Control

Table S3. hCF Doping Experiments Force-Frequency Summary.

	Frequency		1Hz		1.5 Hz		2 Hz		2.5 Hz	
**Measure**	**Group**		**Mean (n)**	**SEM**	**Mean (n)**	**SEM**	**Mean (n)**	**SEM**	**Mean (n)**	**SEM**
**Stress (μN/ mm²)**	Control		27.1 (7)	5.0	21.5 (7)	4.2	16.9 (5)	3.7	7.4 (6)	1.7
	5%	+hCF	104.0 (12)	11.8	95.9 (12)	11.1	71.4 (12)*	9.4	52.5 (11)**	7.5
	10%	+hCF	90.1 (7)	18.1	86.1 (7)	17.9	70.2 (7)	15.0	51.5 (7)	10.9
	15%	+hCF	75.2 (7)	15.5	77.6 (5)	21.6	52.1 (7)	12.6	38.3 (7)	9.3
**V_{up} (μN/s)**	Control		63.4 (7)	21.3	53.5 (7)	20.0	45.7 (5)	15.3	19.2 (6)	8.9
	5%	+hCF	118.2 (12)	25.9	113.7 (12)	25.6	91.6 (12)	21.0	74.3 (11)	16.2
	10%	+hCF	97.2 (7)	28.3	96.1 (7)	27.5	85.9 (7)	24.7	68.6 (7)	19.9
	15%	+hCF	76.9 (7)	23.0	94.5 (5)	27.1	62.9 (7)	21.3	50.8 (7)	17.0
**T50 (ms)**	Control		261.8 (7)	12.7	210.5 (7)**	4.9	173.0 (5)**	5.8	136.6 (6)**	2.3
	5%	+hCF	222.3 (12)	18.2	182.2 (12)**	6.9	159.1 (12)**	5.4	137.5 (11)**	3.2
	10%	+hCF	187.4 (7)	22.5	169.8 (7)	6.4	151.9 (7)	5.6	131.4 (7)**	2.8
	15%	+hCF	162.5 (7)	20.6	174.3 (5)	11.3	165.4 (7)	18.1	131.3 (7)	9.8
**T90 (ms)**	Control		519.7 (7)	17.7	383.6 (7)**	14.4	311.5 (5)**	6.2	237.6 (6)**	7.7
	5%	+hCF	440.7 (12)	22.6	383.9 (12)*	5.8	306.0 (12)**	8.0	253.9 (11)**	3.3
	10%	+hCF	409.6 (7)	34.6	364.6 (7)	11.0	314.2 (7)**	8.5	254.4 (7)**	3.7
	15%	+hCF	455.1 (7)	65.3	378.5 (5)*	30.0	306.6 (7)**	6.7	254.8 (7)**	7.3

	Frequency		3 Hz		3.5 Hz		4 Hz	
**Measure**	**Group**		**Mean (n)**	**SEM**	**Mean (n)**	**SEM**	**Mean (n)**	**SEM**
**Force (μN/ mm²)**	Control		3.2 (2)	0.9	1.7 (3)	0.9	1.0 (1)	N/A
	5%	+hCF	27.6 (8)**	5.3	23.7 (3)**	1.0	1.1 (3)**	0.9
	10%	+hCF	33.5 (7)**	6.9	24.6 (5)**	6.8	13.1 (4)**	5.6
	15%	+hCF	33.6 (6)	7.9	23.5 (6)	5.9*	16.2 (6)**	4.4
**V_{up} (μN/s)**	Control		8.6 (2)	1.5	5.1 (3)	1.7	3.4 (1)	N/A
	5%	+hCF	42.0 (8)*	15.0	39.4 (3)	22.4	8.1 (3)*	3.3
	10%	+hCF	49.8 (7)	13.8	40.4 (5)	13.8	24.4 (4)	9.8
	15%	+hCF	47.3 (6)	13.5	36.7 (6)	11.0	27.1 (6)	8.9
**T50 (ms)**	Control		119.4 (2)**	3.0	96.5 (3)**	1.0	81.9 (1)	N/A
	5%	+hCF	110.5 (8)**	6.9	96.4 (3)**	3.0	85.1 (3)**	3.1
	10%	+hCF	108.8 (7)**	2.4	91.9 (5)**	1.6	78.5 (4)**	1.7
	15%	+hCF	118.6 (6)*	9.2	103.0 (6)**	6.1	88.7 (6)**	5.7
**T90 (ms)**	Control		209.2 (2)**	4.4	171.3 (3)**	9.2	157.8 (1)	N/A
	5%	+hCF	200.0 (8)**	10.1	179.8 (3)**	2.9	145.9 (3)**	3.9
	10%	+hCF	209.9 (7)**	4.3	175.7 (5)**	2.0	149.0 (4)**	3.6
	15%	+hCF	208.2 (6)**	3.3	184.4 (6)**	4.9	159.3 (6)**	2.7

Significant vs. Control	Significant vs. Ctrl and 5% hCF

*P<0.05 and **P<0.01 compared to tissues of same experimental group at 1 Hz pacing

Table S4. p4 v p9 hCF Experiments 1 Hz Mechanics Summary.

Measure	Group		Mean (n)	SEM
**Cross-sectional area (mm^2)**	Control		0.213 (18)	0.028
	P4	+ hCFs	0.081 (18)	0.010
	P9	+ hCFs	0.203 (20)	0.012
**Young's Modulus (kPa)**	Control		1.59 (14)	0.36
	P4	+ hCFs	7.89 (16)	1.74
	P9	+ hCFs	2.47 (16)	0.58
**Maximum active stress (mN/mm^2)**	Control		0.141 (15)	0.031
	P4	+ hCFs	0.227 (18)	0.033
	P9	+ hCFs	0.087 (20)	0.015
**Maximum capture rate (Hz)**	Control		4.13 (16)	0.11
	P4	+ hCFs	4.00 (18)	0.07
	P9	+ hCFs	3.87 (19)	0.10

Significant vs. Ctrl and p9 hCF	Significant vs. p4 hCF

Table S5. p4 v p9 hCF Experiments Force-Frequency Summary.

	Frequency		1 Hz		1.5 Hz		2 Hz		2.5 Hz	
**Measure**	**Group**		**Mean (n)**	**SEM**	**Mean (n)**	**SEM**	**Mean (n)**	**SEM**	**Mean (n)**	**SEM**
**Stress (μN/mm²)**	Control		123.4 (16)	19.1	120.4 (16)	18.7	102.2 (16)	15.2	75.1 (16)*	10.5
	P4	+hCFs	193.0 (18)	25.5	176.4 (18)	21.7	154.4 (18)	18.3	120.2 (18)**	13.9
	P9	+hCFs	76.3 (20)	7.7	74.1 (20)	7.4	65.6 (20)	6.7	50.9 (20)	5.7
**Vup (μN/s)**	Control		156.5 (16)	35.2	152.9 (16)	34.2	138.0 (16)	30.5	110.1 (16)	22.9
	P4	+hCFs	99.1 (18)	12.5	91.5 (18)	10.3	86.1 (18)	9.6	74.8 (18)	8.5
	P9	+hCFs	96.7 (20)	12.4	95.7 (20)	12.1	90.8 (20)	11.1	77.3 (20)	9.4
**T50 (ms)**	Control		154.4 (16)	11.3	148.3 (16)	7.0	148.0 (16)	5.5	133.4 (16)*	5.5
	P4	+hCFs	138.8 (18)	5.5	137.1 (18)	4.5	137.6 (18)	3.8	125.7 (18)	2.9
	P9	+hCFs	136.5 (20)	8.0	137.9 (20)	7.7	136.9 (20)	6.8	123.1 (20)	4.8
**T90 (ms)**	Control		303.1 (16)	26.0	316.5 (16)	12.5	296.6 (16)	5.4	251.2 (16)**	1.9
	P4	+hCFs	291.6 (18)	16.2	311.2 (18)	10.4	292.0 (18)	3.8	244.6 (18)**	3.0
	P9	+hCFs	278.2 (20)	14.5	293.3 (20)	14.8	276.9 (20)	9.1	242.6 (20)*	4.2

	Frequency		3 Hz		3.5 Hz		4 Hz	
**Measure**	**Group**		**Mean (n)**	**SEM**	**Mean (n)**	**SEM**	**Mean (n)**	**SEM**
**Stress (μN/mm²)**	Control		52.5 (16)**	7.1	37.0 (16)**	4.8	16.3 (13)**	1.9
	P4	+hCFs	87.6 (18)**	10.1	63.2 (18)**	7.2	42.6 (16)**	5.5
	P9	+hCFs	36.2 (20)	4.6	28.3 (17)*	4.0	19.3 (15)**	2.3
**Vup (μN/s)**	Control		85.1 (16)*	16.9	65.5 (16)**	13.1	32.2 (13)**	5.6
	P4	+hCFs	61.1 (18)	7.1	48.0 (18)	6.0	33.9 (16)*	4.4
	P9	+hCFs	58.3 (20)	7.7	51.2 (17)	6.7	42.4 (15)	5.4
**T50 (ms)**	Control		113.6 (16)**	2.2	95.6 (16)**	1.6	76.3 (13)**	5.8
	P4	+hCFs	109.9 (18)**	1.9	94.4 (18)**	1.1	80.5 (16)**	1.2
	P9	+hCFs	110.1 (20)**	3.3	94.8 (17)**	2.2	82.2 (15)**	1.9
**T90 (ms)**	Control		206.7 (16)**	2.7	177.5 (16)**	2.3	142.8 (13)**	10.7
	P4	+hCFs	206.9 (18)**	1.7	177.5 (18)**	1.0	156.5 (16)**	1.7
	P9	+hCFs	211.5 (20)**	3.6	177.3 (17)**	1.8	155.8 (15)**	15

Significant vs. Control	Significant vs. Ctrl and p9 hCF	Significant vs. Ctrl and p4 hCF	Significant vs. p9 hCF	Significant vs. p4 hCF

*P<0.05 and **P<0.01 compared to tissues of same experimental group at 1 Hz pacing
